# Optically Tracked Reversible Single-Atomic-Monolayer Redox in Plasmonic Nanocavities

**DOI:** 10.1021/acs.nanolett.6c02438

**Published:** 2026-09-21

**Authors:** Eric S. A. Goerlitzer, Zijia Wu, Angus Crookes, Shu Hu, Rakesh Arul, Sarah May Sibug-Torres, Bart de Nijs, Reshma R. Rao, Mary P. Ryan, Angela Demetriadou, Jeremy J. Baumberg

**Affiliations:** † NanoPhotonics Centre, Department of Physics, Cavendish Laboratory, 2152 University of Cambridge, Cambridge CB3 0US, United Kingdom; ‡ School of Physics and Astronomy, 1724 University of Birmingham, Edgbaston, Birmingham B15 2TT, United Kingdom; § Physics for Sustainable Chemistry Group, Department of Physics, Cavendish Laboratory, 2152 University of Cambridge, Cambridge CB3 0US, United Kingdom; ∥ Department of Materials, 4615 Imperial College London, Exhibition Road, SW72AZ London, United Kingdom

**Keywords:** spectro-electrochemistry, atomic-scale redox, underpotential deposition, NPoM, plasmonics, switching

## Abstract

Controlling redox reactions at the atomic scale is key to enabling next-generation low-power memristive and neuromorphic devices. Ultrathin metallic layers, such as atomic monolayer palladium (Pd), offer tunable redox behavior well suited for switchable components in nanoscale electronics. Here, we present an in situ plasmonic-electrochemical platform that probes redox processes within subnanometer gaps using a nanoparticle-on-mirror (NPoM) geometry. Atomically thin Pd layers are grown by underpotential deposition, and their redox is monitored via surface enhanced Raman spectroscopy, revealing reversible transformations between metallic and dielectric Pd redox states. This confined plasmonic cavity enhances sensitivity to vibrational and electronic changes, allowing nondestructive optical readout of redox switching behaviors. By reducing the active switching volume to the monolayer limit while maintaining precise geometric control, this work establishes a powerful platform for probing redox dynamics at the atomic scale and points toward highly energy-efficient nanoswitches based on redox-active ultrathin materials.

## Graphical abstract

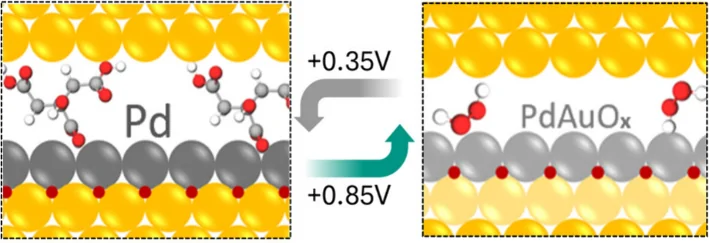

Controlling reduction–oxidation (redox) reactions within ever-smaller active volume is emerging as a promising strategy for developing next-generation memory, logic, and neuromorphic systems. Of particular interest are memristors or resistive-random access memories (RRAMs) in which a nanoscale conductive metal path between an anode and cathode is formed or broken by mobile metal cations, or by the migration of oxygen vacancies.^1,2^ Such resistance switching nanodevices have counterparts in the optical domain, where conductance switching across a metallic gap modifies the optical confinement of plasmon modes.^3–7^ However, their active switching regions typically extend over tens of nanometers or possess poorly controlled geometric precision.

A key challenge for such devices is how to control nanodevice morphologies when their dimensions approach the atomic scale. Ultrathin metallic layers have emerged as frontier materials science, offering unique catalytic, electronic, and magnetic properties that differ fundamentally from those of their bulk counterparts.^8–13^ Their unique redox-active behaviors enable dynamic and reversible transitions between conductive and dielectric redox states, valuable for modulating both electrical conductivity and optical response. However, accessing and manipulating these switching behaviors in a controlled, reversible, and device-compatible manner remains a significant challenge.

Redox dynamics for single atomic monolayers remains largely unexplored, partly because of the difficulty of effectively probing chemical specificity and reversibility. While *in situ* electron microscopy and *ex situ* tomography provide structural insights, their destructive nature and nonambient conditions limit their ability to capture realistic operational redox behavior. By contrast, optical spectroscopy utilizing plasmonic nanostructures offers a nondestructive high-sensitivity platform for probing atomic-scale transformations, because it confines optical fields far below the diffraction limit and enhances local field intensities by several orders of magnitude.^4,14^ The resulting surface-enhanced Raman spectroscopy (SERS) becomes a powerful tool for real-time chemically specific monitoring of redox processes at the atomic scale.^15,16^

Here we explore the ultimate limits when confining the redox switching volume to a single atomic monolayer within a well-defined subnanometre gap. We achieve this using electrochemically gated plasmonic devices that enable direct redox modulation and in situ optical interrogation of Pd atomic monolayers. These are embedded in a nanoparticle-on-mirror (NPoM) geometry which provides precise control of sub-nm gap spacing and operates in a dual role as both plasmonic cavity and localized electrochemical reactor.^17^ We employ underpotential deposition to achieve atomically precise growth of Pd monolayers on the mirror facet and demonstrate their low-energy reversible oxidation to form an interfacial oxide PdAuO_*x*_ within the ultrasmall active volume. This transition dynamically and reversibly alters both the optical response and local electronic environment tracked using SERS in real time under ambient conditions. These findings offer a powerful platform for studying the redox switching mechanism of metal monolayers and suggest promising pathways for developing highly tunable efficient devices at the atomic scale.

To embed a uniform atomic monolayer of Pd within NPoM structures (‘Pd_ML_-NPoM’, Figure 1A), a template-stripping method is first used to produce atomically smooth Au films.^18^ The Pd monolayer (Pd_ML_) is then deposited through underpotential deposition (UPD) in a three-electrode electrochemical cell (Figure S1).^19^ Cyclic voltammetry confirms the process, showing clear UPD and overpotential deposition (OPD) peaks, as well as Pd stripping behavior (Figure 1B), highlighting precise control over Pd monolayer formation (see also Figure S2). The Au nanoparticles (AuNPs) of diameter 80 nm are then drop-cast at low concentration on the films, to form NPoMs which can be individually located (images Figure 1C) and measured.

**Figure 1:**
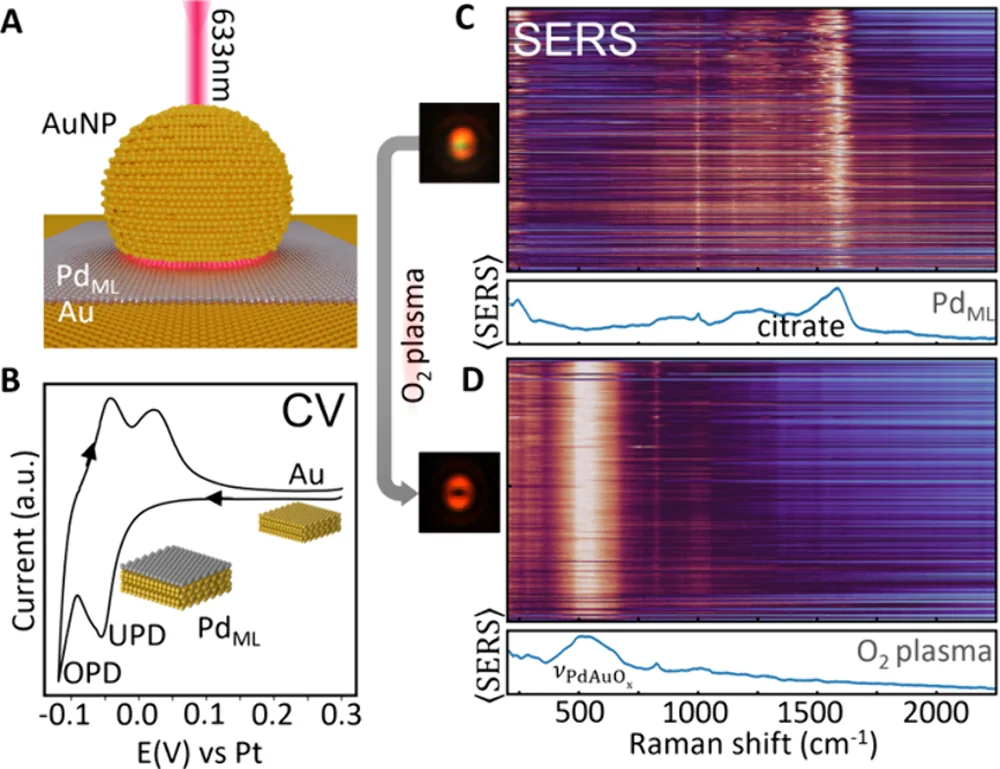
Single Pd monolayer embedded in plasmonic nanocavity. (A) Schematic of NPoM construct. (B) Cyclic voltammetry (CV) of UPD growth of Pd_ML_ on gold. (C, D) SERS from >200 NPs and averaged spectra (C) before and (D) after plasma oxidation. Insets show dark-field images of NPoMs containing Pd_ML_.

Initially, an oxygen plasma clean (O_2_–PC) is used to oxidize the Pd_ML_ (Experimental Methods), forming a AuNP-PdO_*x*_-Au sandwich structure. During oxidation, reactive oxygen species break down ligands and impurities adsorbed on the nanoparticle surfaces, allowing the oxygen to react with the bare metal, thereby forming a stable oxide layer. This follows extensive previous work showing corresponding oxidation of Au in AuNP nanogaps using this method.^20^ The nanogap contents are analyzed by collecting 633 nm-pumped SERS spectra on each NPoM. Statistical analysis of hundreds of NPoM nanogaps shows no detectable metal oxide peaks before plasma treatment, confirming the stability of the Pd_ML_ in air (Figure 1C). The citrate peaks seen around 1600 cm^–1^ are from the initial ligands around the AuNP, which are present in various orientations on the Au.^21^

After 10 min of O_2_–PC, these citrate peaks disappear leaving only minimal residue ∼1100 cm^–1^, corresponding to C–C stretches (Figure 1D). By contrast, a broad pronounced peak appears centered at ∼540 cm^–1^ providing clear evidence of Pd_ML_O_*x*_ formation.^22^ Compared to the measured ∼590 cm^–1^ AuO_*x*_ peak (reported in previous literature,^23^ see also Figure S7), this shifted peak confirms that the oxide formed is predominantly Pd_ML_O_*x*_ on the bottom facet.^23,24^ On flat substrates the weakly observed Pd–OH (750 cm^–1^) peak is also down-shifted from Au–OH (800 cm^–1^). Both these down-shifts are identical for the 1 ML Pd and thick Pd samples, implying minimal Au oxidation accompanies the formation of Pd_ML_O_*x*_. We note oxidizing Au removes all citrate,^23,25^ and with no changes in the oxide peak for increasing plasma oxidation times, this gives no indication that there are contributions to the oxide lines from organics.

X-ray photoelectron spectroscopy (XPS) further confirms this monolayer deposition on flat films. XPS spectra of the Pd 3d line (Figure 2A) at different X-ray photon energies (to probe different depths) demonstrate consistent line shapes and binding energies. These features all originate from the same surface Pd atoms, confirming monolayer formation on top of gold. These Pd 3d peaks appear at higher binding energies than for bulk Pd (335.4 eV, vertical line), further validating its monolayer character. We assign the peak at 335 eV as Au 4d_5/2_ (rather than bulk Pd 3d_5/2_) due to the absence of a paired Pd 3d_3/2_ peak expected at 340.31 eV, and because it vanishes nearest to the surface (for lower photon energies).

**Figure 2:**
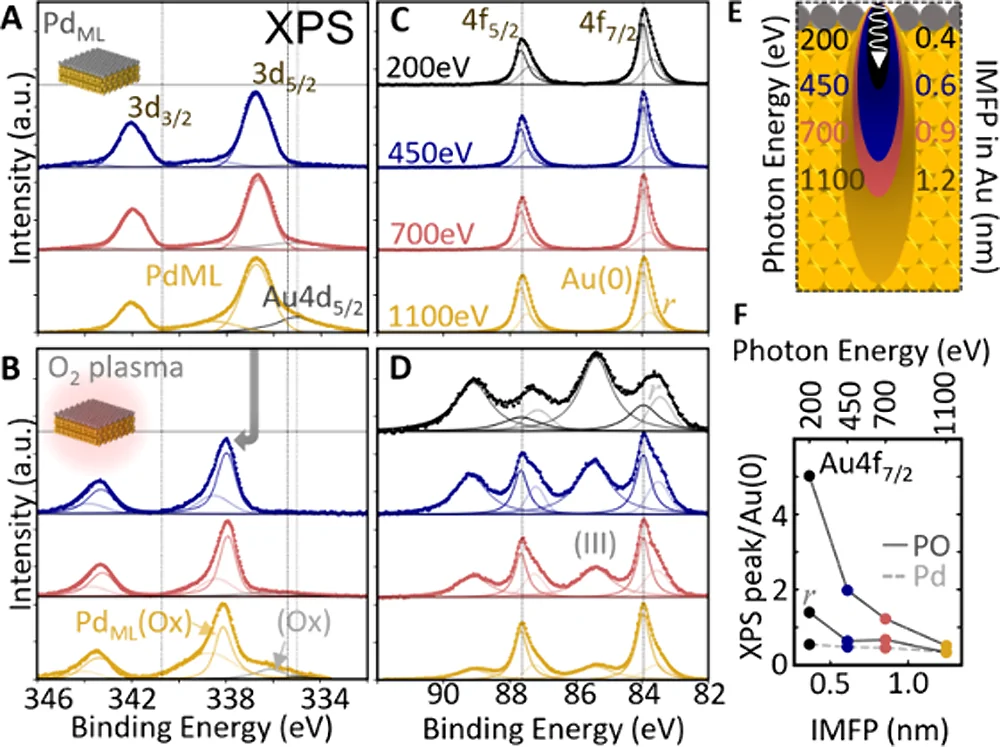
XPS of single Pd monolayer. (A–D) XPS spectra showing intensity vs binding energy at photon energies 200–1100 eV for (A, B) Pd 3d and (C, D) Au 4f core levels, both (A, C) before and (B, D) after oxidation. (E) Sampling depth in bulk Au at each photon energy, set by the inelastic mean free path (IMFP) of the emitted photoelectrons, whose kinetic energy is determined by the photon energy. (F) Au­(III) 4f7/_2_ component ratios vs photoelectron IMFP, before (Pd, dashed) and after (PO, solid) oxidation.

The surface-sensitive Au 4f spectra (Figure 2C, D) with XPS probing depths ranging from 0.4 to 1.3 nm, reveal a distinctive 83.6 eV side peak (*r*) emerging at lower photon energies and thus at the surface (Figure 2E, F). This side peak maintains a stable position with a consistent shift of −0.2 eV from the bulk peak. A comparable shift of −0.2 eV has been documented for monolayer gold deposited on bulk palladium,^26^ supporting our attribution of this side peak to gold atoms in direct contact with the palladium monolayer.

Oxidation of the interface in an oxygen plasma drastically alters the landscape of the observed XPS binding energies. All elemental Pd 3d is completely replaced by new peaks at ∼1 eV higher binding energy (Figure 2B), typical of oxidized states. The Pd oxide peaks appear with identical shape and position for all photon energies, implying it exists as a stable continuous monolayer, rather than formed of islands (which would give mixed peaks). At higher photon energies (deeper penetration), the Au 4d^5/2^ peak is also upshifted from bulk Au, and thus oxidized (gray arrow Figure 2B).

Further understanding is provided by analyzing Au 4f signals. In stark contrast to Pd, depth-dependent peak amplitudes result from oxidized Au near the air interface. At 200 eV, the spectrum is dominated by Au­(III), as well as surface-reconstructed gold (*r*). These samples were kept in ambient conditions for 1 week before measurement, to exclude unstable gold oxide species which decay after 1 day.^27^ This surface Au­(III) must therefore come from formation of a bimetallic monolayer of Au and Pd, in which Au­(III) remains completely stable. This first observation of stable monolayer PdAuO_*x*_ explains the unusual oxide SERS peak which resembles neither pure metal oxide. We note that both electrochemical- and plasma-oxidation of Au forms Au­(III)_2_O_3_, as identified from both DFT analysis of the SERS peaks and from XPS.^20,23^ We emphasize that while direct XPS characterization inside individual NPoM nanogaps is not experimentally feasible, the planar regions undergo oxidation at the same time, and the nanogap chemistry is independently supported by the corresponding SERS, DF and CV. Thus, we corroborate that oxidation proceeds identically for both planar and nanogap regions, and thus expect the same stoichiometry.

To observe dynamic redox switching of this Pd_ML_, an alternative oxidation technique to vacuum-based plasma treatment is required. We thus utilize a spectro-electrochemical cell for recording in situ reversible redox transformations at ambient conditions (Figure S3). Initially cyclic voltammograms for bulk Au, Pd, and a Pd monolayer on Au are compared to reveal distinct shifts in reduction peak positions (Figure 3A). A stable narrow Pd_ML_O_*x*_ reduction peak is observed, with typically broad Pd oxidation peak from its different oxidation states.^28^ The absence of Au oxidation and reduction peaks at +0.7 V and +0.85 V confirms that the Pd monolayer completely protects the underlying Au, indicating full Pd monolayer coverage. This has previously been also confirmed by using nanogap molecules whose SERS spectrum changes depending on the metal to which they are bound.^19^ The observed +0.1 V increase in the reduction potential compared to PdO_*x*_ (on bulk Pd) for Pd_ML_O_*x*_ in the Pd monolayer on Au arises from hybridization between Au and Pd electronic structures, as well as surface strain induced by lattice mismatch. These effects have previously been shown to collectively displace the metal *d*-bands, creating a surface with enhanced catalytic activity compared to bulk Au or Pd.^29^ This also confirms that only 1 ML of Pd is formed on the Au, and that the nanoparticle Au facet is not oxidized.

**Figure 3:**
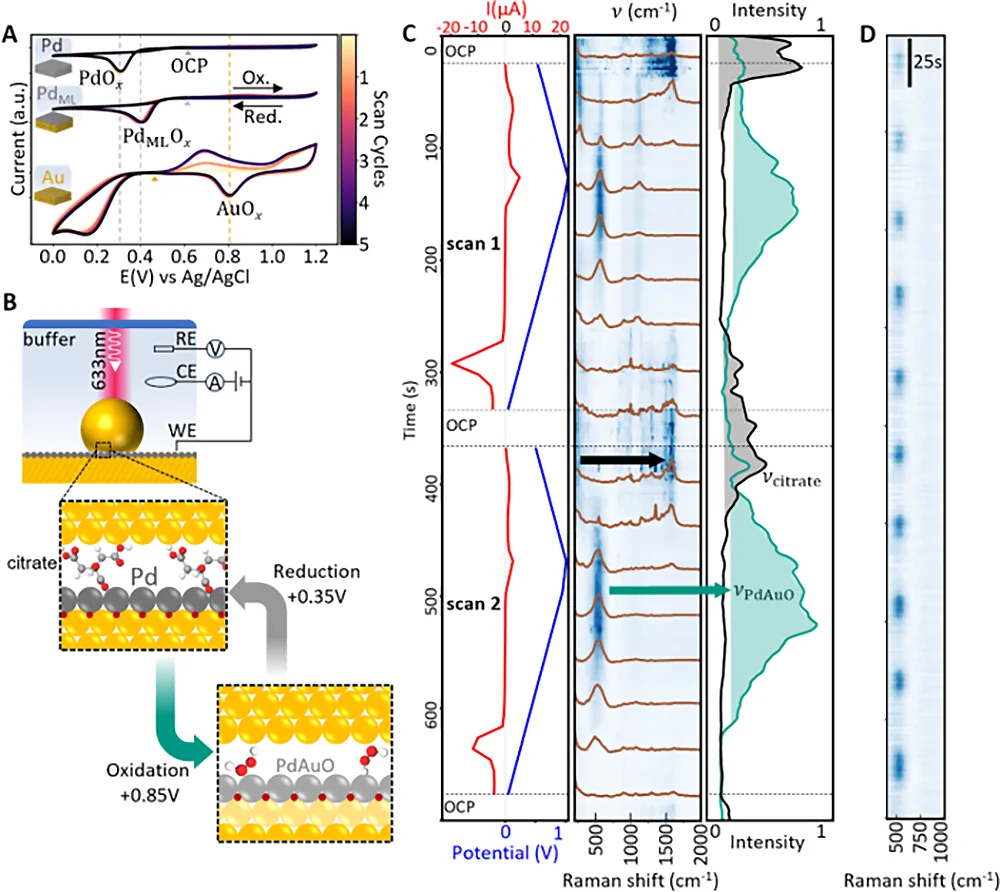
Electrochemical switching of single Pd monolayer. (A) Cyclic voltammograms of Pd, Au, and Pd_ML_ samples when cycling from 0 to 1.2 V (vs Ag/AgCl). (B) Electrochemical cell for single NPoM SERS, with schematic showing inferred Pd redox switching. (C) In situ EC-SERS of single NPoM initially containing Pd_ML_, over 2 cycles from 0 to 1 V (vs Ag/AgCl) with SERS spectra showing switching, tracked via extracted PdAuO_*x*_ and citrate peak areas (right, arrows). (D) Reversible multigap switching in SERS spectra of Au@Pd NP MLaggs over 10 cycles from 0 to 1 V­(vs Ag/AgCl). Scan rate 50 mV/s in 50 mM PBS.

Unlike plasma cleaning, which relies on reactive oxygen surface collisions, electrochemically induced oxidation occurs within seconds (even in nanogaps), due to the direct application of potential to the electrode surface.^23^ Potential is thus applied to the NPoM Au mirror substrate utilizing it as a working electrode (Figure 3B), while to allow for high NA collection of light from above, the distance between coverslip and substrate is minimized, leaving only a ∼300 μm electrolyte layer in between.

By adjusting the anodic and cathodic potential range at the Au-based SERS substrate, we achieve precise control over oxidation, reduction, and desorption of adsorbates. Simultaneous time-series SERS spectra for AuNPs on a Pd_ML_-coated Au mirror, and cyclic voltammetry, are recorded (Figure 3C) as potential is swept between +1 and 0 V at 5 mV/s for two cycles in 50 mM potassium phosphate buffer (PBS, pH 7.0). Immediately after applying +0.5 V, the citrate peak intensity drops while the PdAuO_*x*_ intensity grows, maximizing at +1 V. Upon subsequent reduction, the metal oxide peak completely disappears leaving a more complex set of citrate lines (possibly modified by decomposition products). We note that citrate has multiple binding configurations on Au.^21^ This alternation between citrate and PdAuO_*x*_ signals repeats in subsequent scans, confirming that PdAuO_*x*_ formation is repeatable, even for ultrathin Pd films (cycling a further 7 times is shown in Figure S4). Although atomic-scale defects or local rearrangements at the Pd_ML_/Au interface during individual redox events cannot be excluded, no technique currently allows direct structural characterization inside the active NPoM nanogap under operating conditions. Hence spectroscopy gives key information. We note these changes are rapid (<1s, faster than can be resolved in the current setup, even applying step potentials) and require low switching energy. For a single NPoM gap, the active area is *A*_gap_ ≈ 300 nm^2^(for cavity facet diameter of 20 nm). From monolayer Pd coverage on Au(111), the number of Pd atoms switching in the gap *N*_Pd_∼3000. Assuming *n* = 3 electrons transfer per Pd atom during oxidation at characteristic switching potential *V*_*s*_ ≈ 0.8 V, the transferred charge is *Q*_gap_ = *neN*_Pd_ ≈ 1.4 × 10^–15^ C, and the energy cost *U*_gap_ ≈ *Q*_gap_ *V*_*s*_ ∼ 1 fJ. We stress that this is an intrinsic thermodynamic lower bound on the charge-transfer cost within a single gap which informs a scaling argument on the potential benefits of confining the active switching volume to a monolayer. While for smaller facets,^15^ this switching energy could be reduced 10-fold, we note other contributions may also be involved.

Further experiments on many thousand nanogaps in bulk solution are performed to confirm the observed oxide vibrational peaks (Figure 3D, see also Figures S5–S6). These use nanogaps between 1 ML Pd-coated 80 nm Au NPs (Au@Pd NPs) assembled into monolayer aggregates (MLaggs).^20,23,30,31^ Extensive prior work has shown their identical nanogaps are well-coupled to incident light. The observed switching behavior in these MLaggs is consistent with the individual Pd_ML_-NPoM.

To better understand the nanoscale materials changes in the plasmonic gap, we use dark-field scattering spectroscopy to track the plasmonic resonance.^15^ The NPoM dominant (*lm*) = (10) spectral peak λ_10_ is sensitive to the nanoparticle diameter as well as the gap size and refractive index. Because AuNPs are not identically sized, it is crucial to record several hundred NPoM dark-field spectra. The resulting λ_10_ histograms (Figure 4A) show surprisingly small shifts before/after oxidation, redshifting ∼10 nm from λ_10_= 850 nm. Previous work shorting out such nanogaps with metal^32^ shows that much larger shifts would be expected if the entire nanogap becomes conducting. Our data thus shows that a conducting short is not fully formed upon reduction.

**Figure 4:**
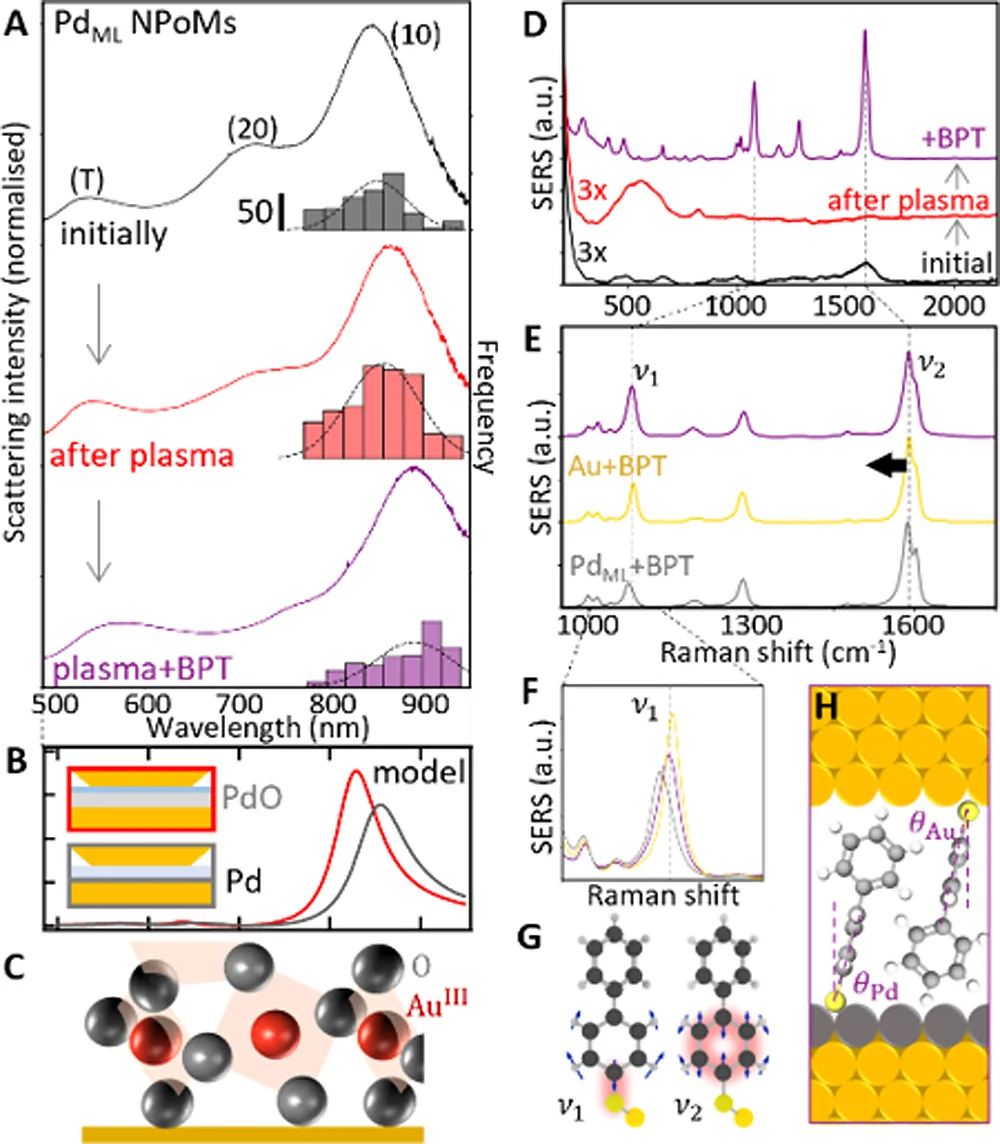
Spectroscopy of plasmonic nanocavities containing a single Pd monolayer. (A) Dark-field scattering spectra of 1 ML Pd NPoMs as initially prepared, after plasma oxidation, and after soaking in BPT. For each sample, several hundred NPoMs are measured (*N* > 200), and a histogram plotted of the dominant^10^ peak position. Spectra shown are average of modal bin. (B) Simulation for (gray) 1 ML Pd capped with 1 nm of *n* = 1.5 dielectric, and (red) 1 nm PdO in nanogap capped by 0.3 nm graphitic carbon. (C) Nominal crystal structure of Au­(III)_2_O_3_ (see text). (D) SERS spectra for cases in (A). (E, F) SERS spectra after plasma, compared to after adding BPT to NPoM without Pd (yellow) and to initial PdML (gray). (G) Dominant BPT vibrational modes from DFT. (H) Schematic of inferred BPT intercalation.

Electromagnetic modeling is thus used to account for these spectral positions, using tabulated refractive index data for the layers.^33,34^ Such modeling has previously accurately accounted for NPoM scattering peaks.^15^ For the reduced state, this reveals that when Pd metal fully fills the gap between AuNP and Au mirror, the observed mode should be at 634 nm (with fields localized in the crevice at the AuNP base). To reach a longer wavelength resonance, an additional thin dielectric gap needs to be included, which is indeed also suggested by our SERS measurements with the observation of citrate SERS peaks. In simulations we thus add a 1 nm-thick dielectric layer (*n* = 1.5) of citrate on the Pd, which shifts the resonance to 820 nm, as observed (Figure 4B). Interestingly, after each cycle the SERS shows that citrate returns into the nanogap, as also for other organic molecules.^23^ For the oxidized state, simulating a PdO dielectric spacer shows that it requires a thickness of ∼1.3 nm to match the observed spectral position, thicker than expected from oxidizing 1 ML Pd. We speculate that the citrate layer is converted into a graphitic carbon layer above the PdO during oxidation, with oxygen atoms transferring into the PdAuO_*x*_, which produces a similarly shifted λ_10_ (Figure 4B).

We thus examine the accessibility and binding in the Pd_ML_O_*x*_ nanogaps by subsequently immersing the oxidized Pd_ML_-NPoM in a solution of thiolated BPT (biphenyl-4-thiol). The oxidized Pd monolayer immediately undergoes chemical reduction, evidenced by the disappearance in SERS of the metal oxide peak at ∼540 cm^–1^ (Figure 4D). This is consistent with previous studies showing that thiols can reduce metal oxides upon binding.^35^ The dark-field (DF) NPoM scattering spectra also further redshift by Δλ ≃ 25 nm compared to the oxidized PdML (Figure 4A, purple), suggesting an increase in gap refractive index or reduction in gap size (increases in facet size can also redshift λ_10_). The SERS spectra of the BPT-functionalized NPoMs now show prominent molecular vibrations, including the ν_1_peak ∼1080 cm^–1^ (C–S stretch, Figure 4G) and the ν_2_ peak ∼1600 cm^–1^ (aromatic C=C stretch).

The positions of these peaks are sensitive to the metal species they bind to. The ν_1_ position of BPT on Au (Figure 4E, F yellow) is 30 cm^–1^ larger than when this Au is coated by the Pd_ML_ (gray). After the Pd_ML_ has been oxidized and then chemically reduced, the ν_1_ position (purple) is between these, 20 cm^–1^ larger than initially, while its line width is unchanged. Pd, with its higher electron affinity and more accessible *d*-orbitals compared to Au, forms stronger covalent bonds with thiol sulfur atoms, leading to a more rigid and electronically coupled metal–molecule interface. The shifts thus suggest a hybrid chemical environment within the cavity, consistent with a mixed Au–S and Pd–S bonding configuration (Figure 4H). In all cases, the thiol is seen to rapidly penetrate the nanogaps, confirming that it retains a molecular layer in all redox conditions.

We now consider the redox atomic restructuring embedded in these nanogaps. While XPS and CV measurements on the planar metal surfaces show that the atomic monolayer of Pd undergoes redox reversibly, the optical measurements (which solely come from the ∼5 nm-wide hotspot in the nanogap) show comparable reconstructions when molecules and Au are also on top. While the strongly oxidizing plasma also co-oxidizes some Au within one atomic layer of the Pd, under electrochemical cycling the absence of Au Faradaic features shows the underlying facet remains unchanged so the oxidized phase touches both metals, but the redox-active volume remains one atomic layer. We emphasize that the structure of Au_2_O_3_ from both plasma and electrochemical oxidation is predicted to be an open 4-fold plaquette structure (Figure 4C), which agrees with the observed SERS line shape and DFT.^23,25^ We thus suggest that the PdAuO_*x*_ retains this coordination since the SERS and CV are similar in AuO samples. Exact atomic positions of this oxide lattice will be shifted by its Au substrate support. Such porous oxides explain the observations that residual citrate as well as the thiols can diffuse into and out of the nanogaps during redox, which may be also correlated with the Pd reduction. We emphasize that although the extreme confinement of light in such nanocavities gives significant insights, it remains challenging for alternative methods to verify directly the atomic structures embedded deep within these nanogaps.

Within this framework, the plasmonic nanogap platform provides a route to interrogate and harness redox-selective switching at the smallest accessible scales at ambient conditions. The persistence of highly repeatable switching despite unavoidable atomic-scale heterogeneity highlights the robustness of this redox mechanism under realistic device conditions. The 10–100 fold reduction in volume within these switching devices gives a corresponding reduction in switching energy, which is of interest for next generation transformer-architecture devices that are currently bottlenecked by large energies as they scale. We also demonstrate that it is important to consider the fate of molecules around these metallic junctions, since they can persist for many cycles. This work paves the way toward developing switching architectures operating with ultrasmall volumes and low energies. We note also a wide range of alternative metals can be similarly incorporated as atomic monolayers,^19^ for additional modulation functionalities.

## Supplementary Material



## Data Availability

All the data in the current study are available from Cambridge Apollo repository, DOI assigned upon publication. All details of materials are provided in the Supporting Information, Experimental Methods. Reasonable requests for materials may be made to the authors. No specific code was created for analysis.

## Notes

Experimental methods Suppl. Figure 1: Electrochemical cell for electrochemical deposition. Suppl. Figure 2: Cyclic voltammograms of Au with different coverages of Pd. Suppl. Figure 3: Optical–electrochemical cell for in situ measurements. Suppl. Figure 4: In situ SERS of PdAuO_*x*_ single NPoM repeated switching. Suppl. Figure 5: In situ SERS of Au@Pd NPs MLaggs with NaNO_3_ repeated switching. Suppl. Figure 6: In situ SERS of Au@Pd NPs MLaggs with NaCl repeated switching. Suppl. Figure 7: Oxide peak position comparisons. (PDF)
