# Supplementary material for: Optically Tracked Reversible Single-Atomic-Monolayer Redox in Plasmonic Nanocavities
Source: Nano Lett. 2026 Sep 21;26(39):13272–8. doi: 10.1021/acs.nanolett.6c02438 (PMC13651603; doi:10.1021/acs.nanolett.6c02438)
Supplement: Supplementary file 1 [file nl6c02438_si_001.pdf]

Supplementary Information

Optically-tracked Reversible Single-Atomic-Monolayer Redox in Plasmonic Nanocavities

Eric S A Goerlitzer[1†], Zijia Wu[1†], Angus Crookes[2], Shu Hu[1], Rakesh Arul[1], Sarah May Sibug-Torres[1], Bart de Nijs[3], Reshma R Rao[4], Mary P Ryan[4], Angela Demetriadou[2], Jeremy J Baumberg[*1]

[1] NanoPhotonics Centre, Dept. of Physics, Cavendish Laboratory,
University of Cambridge, Cambridge, CB3 0US, UK
[2] School of Physics and Astronomy, University of Birmingham, Edgbaston, Birmingham B15 2TT, UK
[3] Physics for Sustainable Chemistry Group, Dept. of Physics, Cavendish Laboratory, University of Cambridge, Cambridge, CB3 0US, UK
[4] Department of Materials, Imperial College London, Exhibition Road, SW72AZ, London, UK

*e-mail: jjb12@cam.ac.uk

Supplementary Information:

Section 1: Experimental methods

Section 2: Supplementary figures

Suppl. Fig. 1 | Electrochemical cell for electrochemical deposition 4
Suppl. Fig. 2 | Cyclic voltammograms of Au with different coverages of Pd 4
Suppl. Fig. 3 | Optical–electrochemical cell for in situ measurements..................... 5
Suppl. Fig. 4 | In situ SERS of $PdAuO_x$ single NPoM repeated switching..................... 5
Suppl. Fig. 5 | In situ SERS of Au@Pd NPs MLaggs with $NaNO_3$ repeated switching..................... 6
Suppl. Fig. 6 | In situ SERS of Au@Pd NPs MLaggs with NaCl repeated switching 7
Suppl. Fig. 7 | Oxide peak position comparisons..................... 8

Section 1: Experimental methods

Chemicals.

Gold (III) chloride trihydrate ($HAuCl_4$, ⩾ 99.9%), palladium (II) chloride ($PdCl_2$, 99%), sulfuric acid (H_2SO_4, 95.0-98.0%), citric acid (99.5%), ascorbic acid (AA, ⩾ 99%) and sodium nitrate ($NaNO_3$, ⩾ 99%), sodium citrate tribasic dihydrate (⩾ 99%) are purchased from Aldrich-Merck. NaCl (≥ 99%), K_2HPO_4 (⩾ 98%) and KH_2PO_4 (⩾ 98%) are from Alfa Aesar and chloroform from Honeywell. 80 nm AuNPs are purchased from BBI Solutions. All chemicals are used without further purification. Milli-Q water (resistivity 18.2MΩ·cm at 25°C) is used in all experiments. All glassware and stir bars are cleaned with aqua regia, piranha solution, and rinsed with Milli-Q water prior to use.

Synthesis of Au@Pd nanospheres.

1.2mL 1%$HAuCl_4$ is diluted in 100mL ultrapure water and boiled in a round bottom flask while stirring with condensate reflux. 1ml 1% sodium citrate is then quickly injected into the flask, giving an average AuNP diameter of 76 nm. Au NPs can also be synthesized using a multi-step approach to enable better morphology control, according to published procedures(*1*). Au@Pd NPs are prepared by adapting established approaches from the literature with slight modifications(*2-5*). 10mL as-synthesized Au NPs and 12μL 1% AA are added to a round bottom flask and kept in an ice bath for 30mins. 133μL 1mM H_2PdCl_4 is slowly injected into the flask while stirring. The excess AA ensures complete reduction of H_2PdCl_4 and its subsequent deposition onto the surface of Au nanoparticles, while the amount of H_2PdCl_4 determines the extent of Pd coverage on the Au nanoparticle surface. The reaction mixture is left overnight, resulting in the formation of a Pd monolayer shell around the Au cores. The shell thickness can be adjusted by varying the amount of H_2PdCl_4 added.

Monolayer Pd electrodeposition.

Monolayer Pd is deposited on ultra-smooth planar Au films using electrochemical underpotential deposition(*6*). The Au substrate is immersed in the electrolyte of 0.1M H_2SO_4 and 1mM H_2PdCl_4 , with Au and Pt wires serving as counter and pseudo-reference electrodes, respectively. A linear voltage sweep is applied from 0.2 to 0.02V *vs* Pt at a scan rate of 1mV s^{-1} to reduce Pd^{2+} species. The distinct reduction peak at approximately 0.05V *vs* Pt, prior to the onset of overpotential deposition (<0.01 *vs* Pt), confirmed the formation of a single Pd atomic layer on the Au surface.

NPoM sample preparation.

Gold substrates with an exceptionally smooth surface are fabricated using the template-stripping technique(*7*). Commercial 80nm BBI Au NPs and synthesized Au@Pd NPs are each diluted at 1:3 with analytical water. 25 μL diluted NPs are drop-cast on top of this gold substrate, followed by 5 μL 1mM $NaNO_3$. These substrates are then rinsed with analytical water immediately and dried with nitrogen gas afterwards.

Monolayer aggregate preparation.

In an Eppendorf tube, 500 mL of as-synthesised Au@Pd NPs are combined with an equal volume of chloroform to create MLaggs SERS substrates(*8, 9*). The Au@Pd NPs begin to aggregate after addition of 150 μL of 1 mM NaCl or $NaNO_3$, which is facilitated by vigorous stirring for 1 min. Following this, the aggregates are permitted to consolidate at the aqueous-organic interface. The aqueous supernatant is replaced with fresh DI water to remove excess ligands and salts. This cleansing procedure is repeated three times. The aggregates are subsequently transferred by carefully reducing the volume of the aqueous phase to approximately 5 μL and depositing the droplet onto a pre-cleaned FTO glass slide. The MLaggs is air dried, rinsed with DI, and desiccated with compressed N_2 after being deposited.

In situ electrochemical cell setup.

A specially designed three-electrode 3D-printed cell is used for in-situ electro-optical measurements (Fig.S1). NPoM samples are used as the working electrode, a platinum wire (Alfa Aesar) as counter-electrode and Ag/AgCl (3 M KCl, eDAQ ET072, Alvatek) as reference electrode. The electrolyte is 0.1M potassium phosphate buffer. The cell is sealed with a $20 \times 20 \times 0.17\ mm^3$ glass coverslip. Electrochemical measurements are recorded on an Autolab PGSTAT204 (Metrohm). Measurements for MLaggs are performed by adapting established approaches from literature(*10*). The cell is sealed and mounted onto the stage of an inverted Raman set-up using custom 3D-printed holders and bases.

Scattering and SERS measurements.

All SERS and scattering spectra measurements of NPoM samples are performed in a home-built confocal Raman microscope, described in detail in previous work(*11*). The system enables automated detection of individual nanoparticles, allowing statistical spectroscopic analysis over large sample sets, as well as an active re-centering function for stage movement to mitigate misalignment and signal fluctuations arising from electrolyte flow. For both scattering and SERS measurements the integration time is 1 s, and all SERS data are collected using a 633 nm excitation laser. SERS measurements for MLaggs are performed utilizing a custom-made Raman setup equipped with a 785 nm diode laser (Matchbox) operating at a power level of $\leqslant 1$ mW with 1 s integration time, details can be found in previous work(*10*).

Synchrotron X-ray photoelectron spectroscopy measurements.

X-ray photoelectron spectroscopy (XPS) is conducted at the Diamond Light Source synchrotron facility (Oxfordshire, UK) on the 109 beamline, using soft X-ray photon energies of 200, 450, 750, and 1100 eV. These correspond to photoelectron kinetic energies with inelastic mean free paths (IMFPs)/escape depths of approximately 0.39, 0.63, 0.88, and 1.24 nm, respectively. Spectra are processed with CasaXPS (www.casaxps.com), and binding energies are calibrated against the Au $4f_{7/2}$ peak at 83.96 eV and the Au $4d_{5/2}$ peak at 335 eV. Survey scans showed only trace levels of carbon and possible oxygen, with no other elements detected.

Oxygen Plasma treatment of Pd_{ML}.

To oxidise the Pd_{ML} and remove the remaining citrate surfactant from the BBI AuNPs, all surface ligands are stripped with oxygen plasma cleaning using 90% RF power and 30 cm^3 STP min^{-1} (Henniker Plasma, HPT-100) for 10 min.

Section 2: Supplementary figures

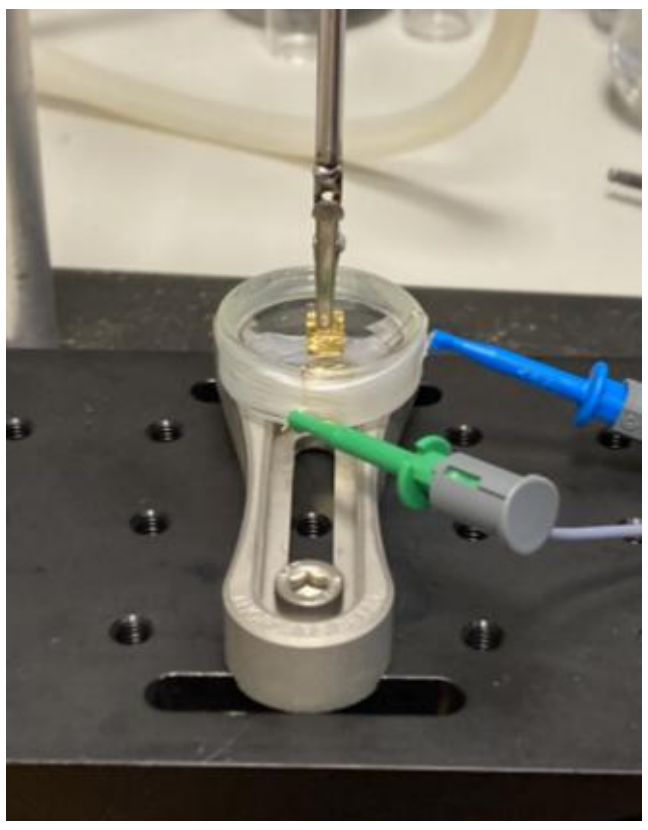

Suppl. Fig. 1 | Electrochemical cell used for electrochemical deposition. A gold substrate was employed as the working electrode (WE) and secured using a stainless-steel clamp. A gold wire served as the counter electrode (CE), connected via the green lead, and a platinum wire functioned as the reference electrode (RE), connected via the blue lead. All electrodes were immersed in the electrolyte within a custom electrochemical cell mounted on a mechanically stable base during deposition.

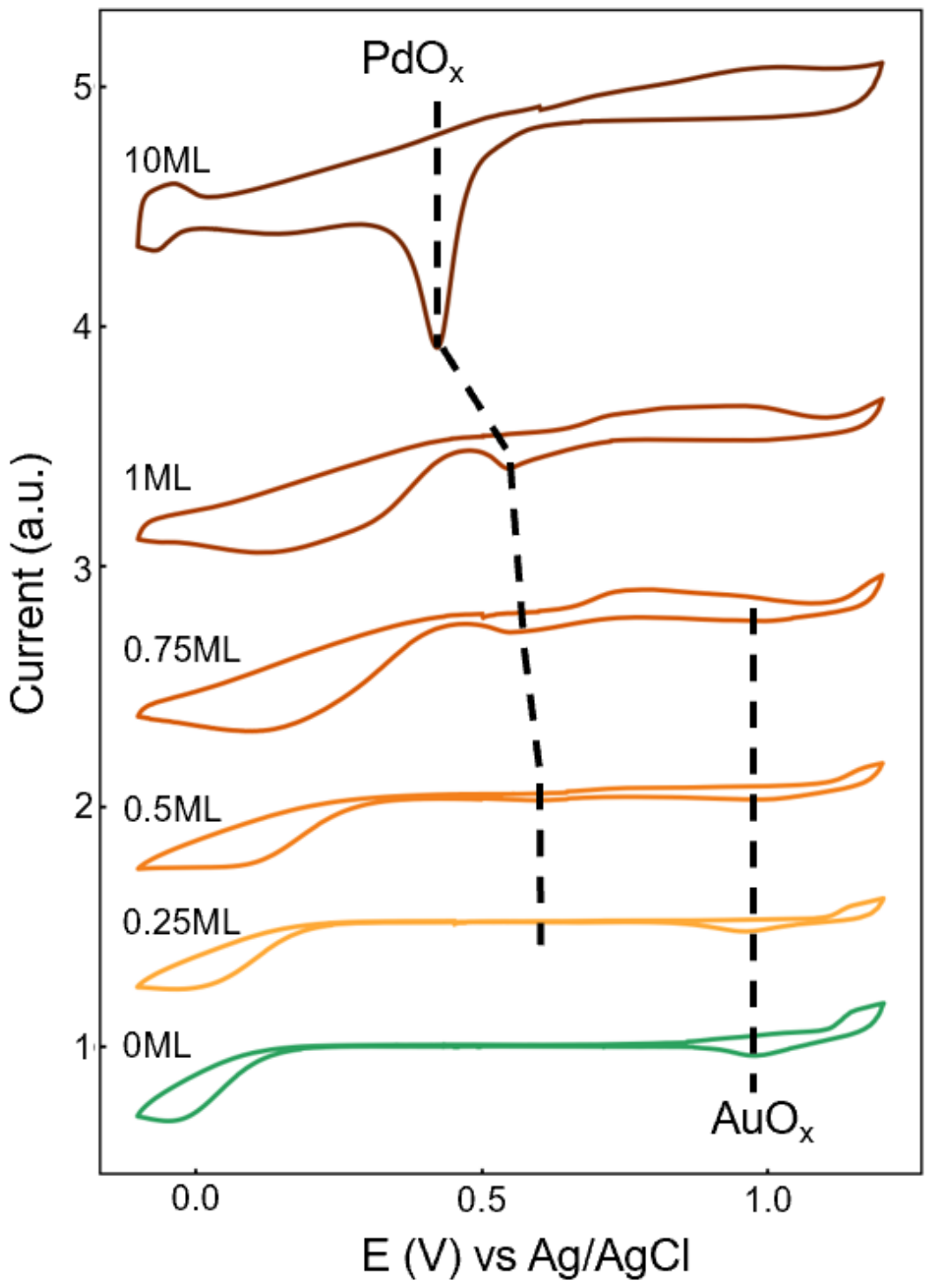

Suppl. Fig. 2 | Cyclic voltammograms of Au with different coverages of Pd. Coverages of Pd on Au mirror are 0, 0.25, 0.5, 0.75, 1 and 10 ML. (bottom to top) (Scan rate = 25 mV s^{-1}, 0.1 M H_2SO_4). A gold wire and Ag/AgCl are used for counter and reference electrodes, respectively.

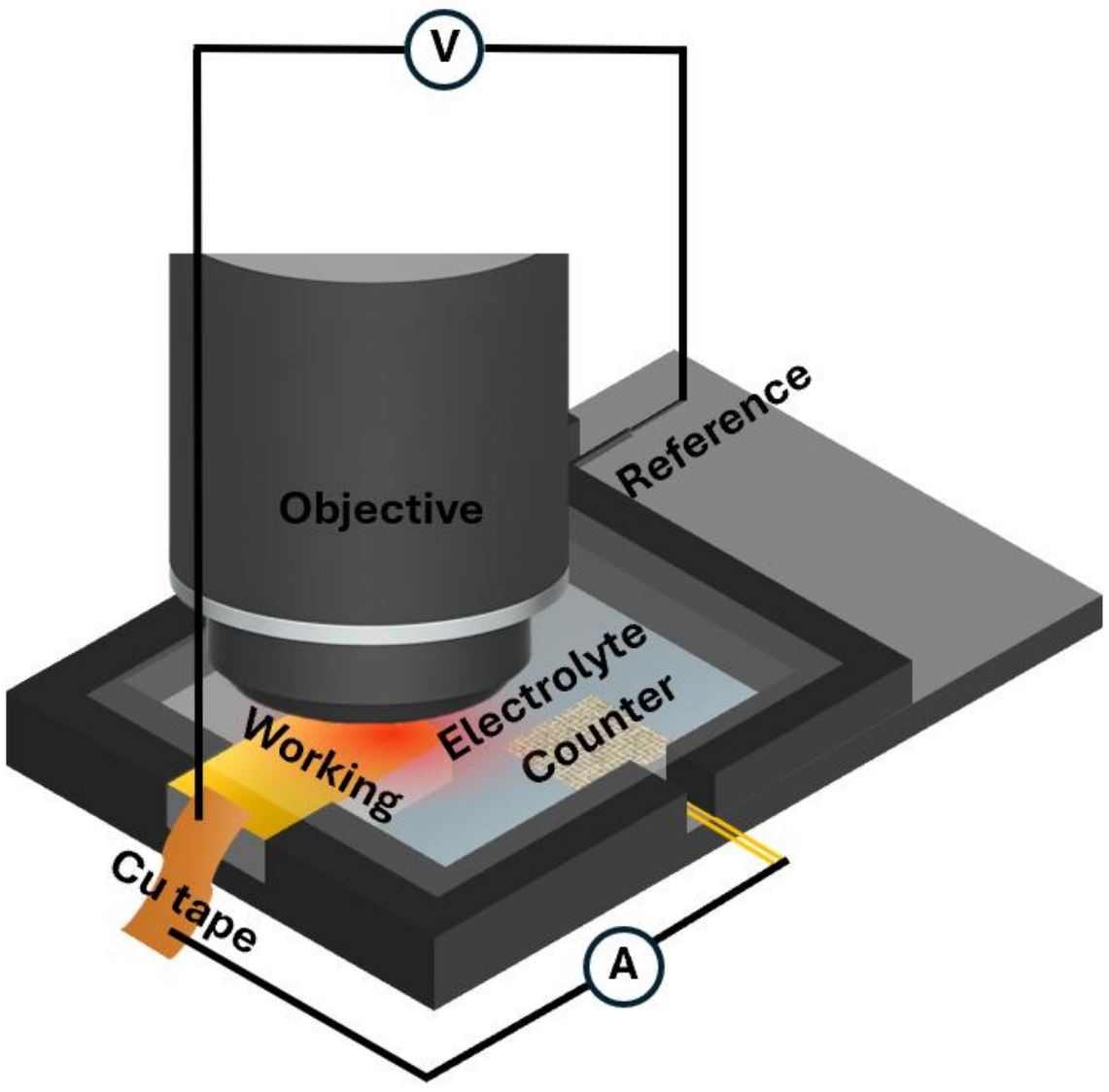

Suppl. Fig. 3 | Schematic of the optical–electrochemical cell for in situ measurements. A gold substrate coated with a monolayer of Pd served as the working electrode (WE) and was positioned beneath the microscope objective. The WE was connected to a potentiostat via copper tape for electrical contact. A cover slip was placed over the substrate to seal the cell, leaving only a thin electrolyte layer in between. A gold wire and a Ag/AgCl electrode were used as the counter electrode (CE) and reference electrode (RE), respectively.

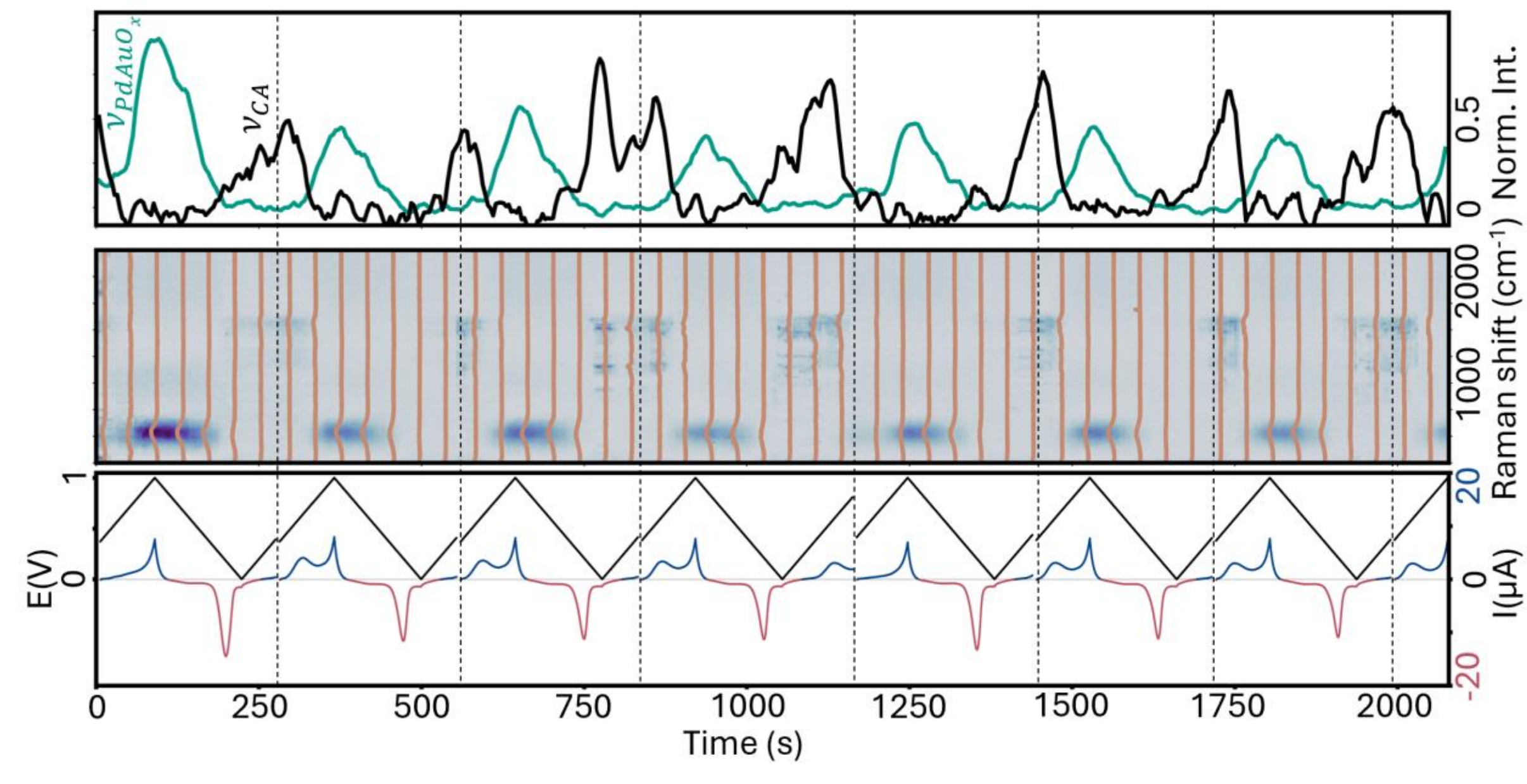

Suppl. Fig. 4 | In situ EC-SERS of $PdAuO_x$ repeated switching. 7 switching cycles of a single Pd_{ML} NPoM from 0 to 1V (*vs* Ag/AgCl) (bottom) with SERS spectra (middle) showing switching, tracked via extracted $PdAuO_x$ and citrate peak areas (top).

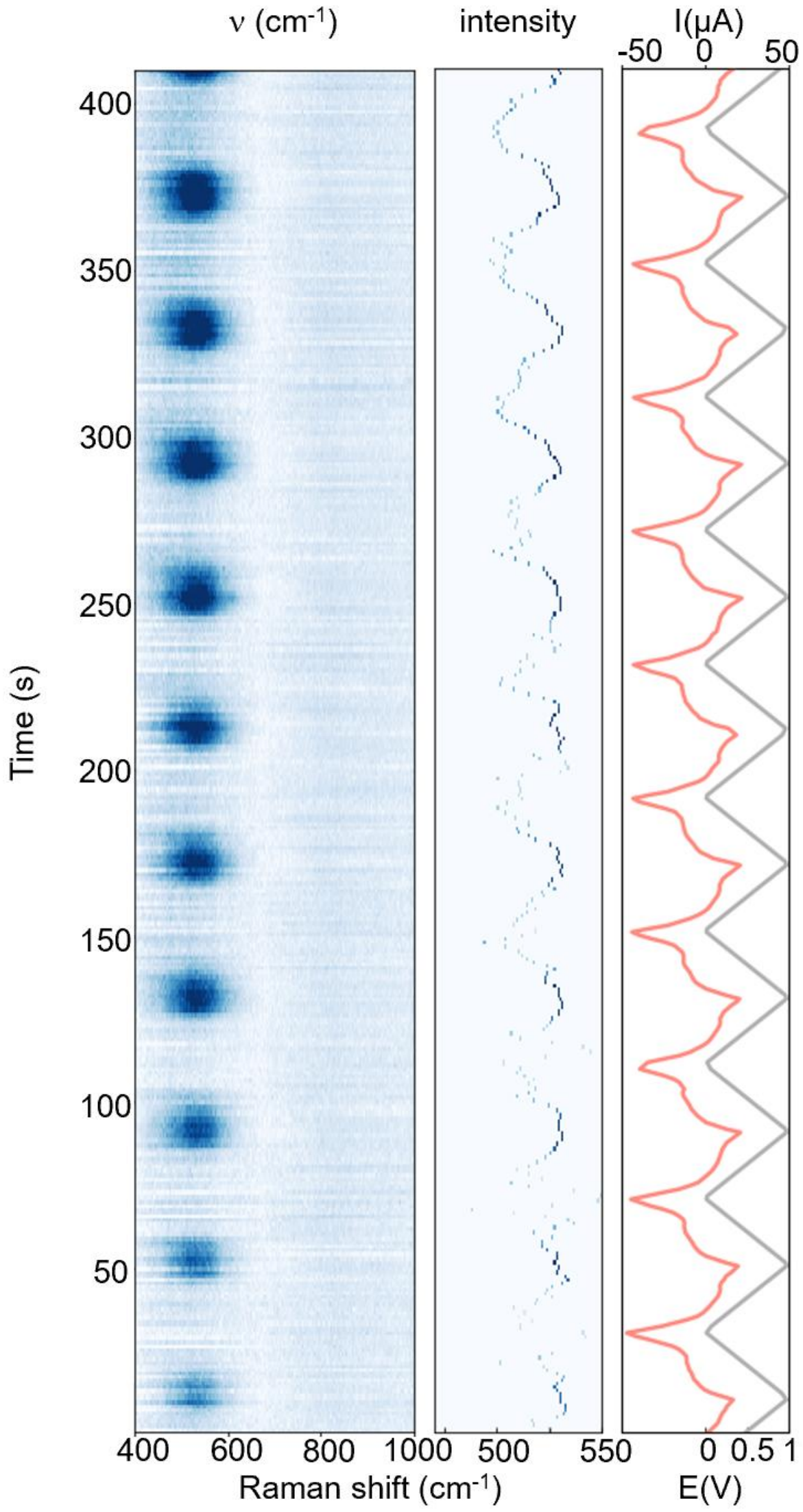

Suppl. Fig. 5| In situ SERS of Au@Pd MLagg repeated switching. 10 switching cycles of Au@Pd MLagg from 0 to 1V (*vs* Ag/AgCl) (right) at 50 mV/s in 50mM PBS with SERS spectra (left) showing switching behaviour, with corresponding peak center position and relative intensity (darker symbols indicate higher intensity)(middle). Nanoparticles were aggregated with $NaNO_3$.

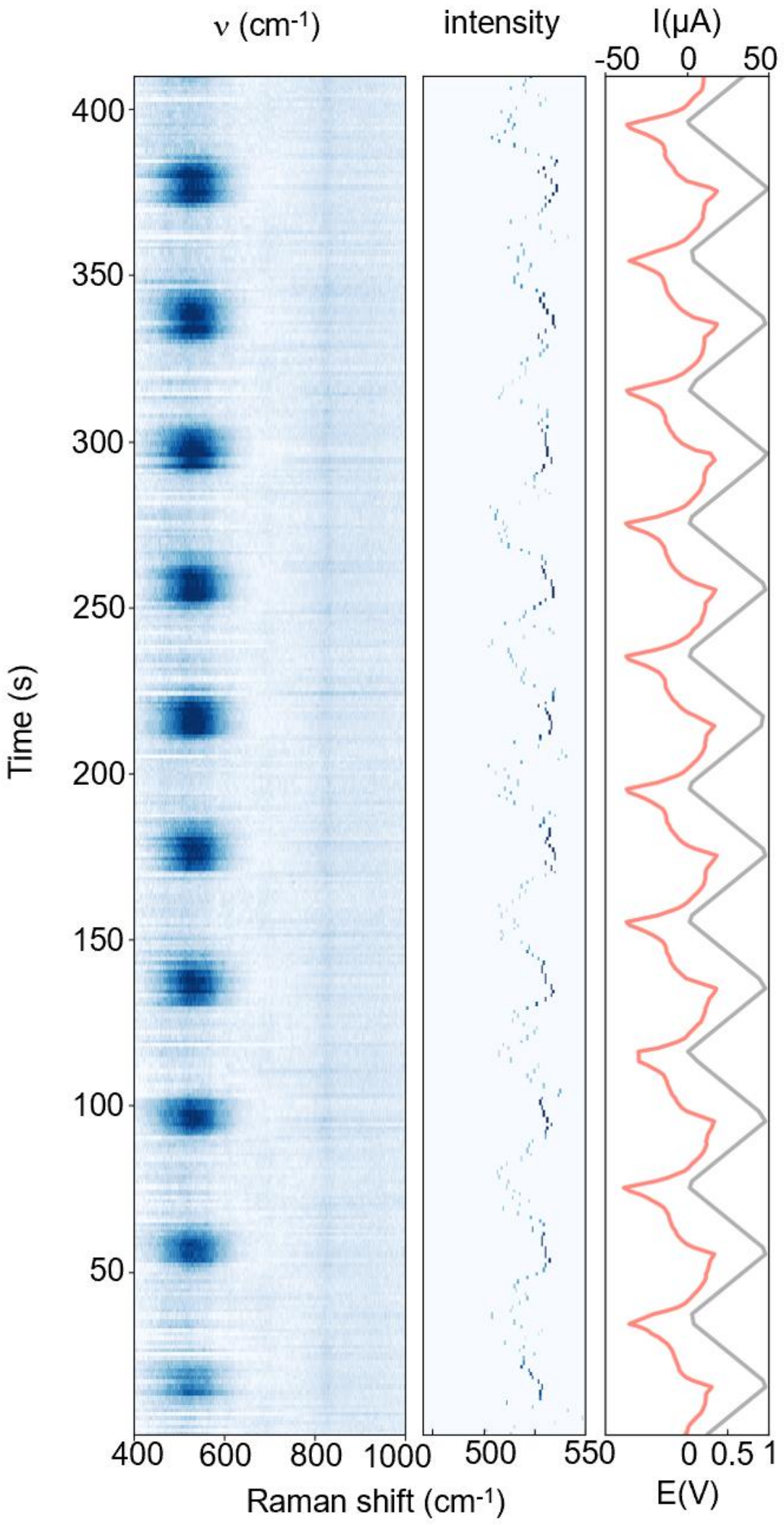

Suppl. Fig. 6| In situ SERS of Au@Pd MLagg repeated switching. 10 switching cycles of Au@Pd MLagg from 0 to 1V (*vs* Ag/AgCl) (right) at 50 mV/s in 50mM PBS with SERS spectra (left) showing switching behaviour, with corresponding peak center position and relative intensity (darker symbols indicate higher intensity)(middle). Nanoparticles were aggregated with NaCl.

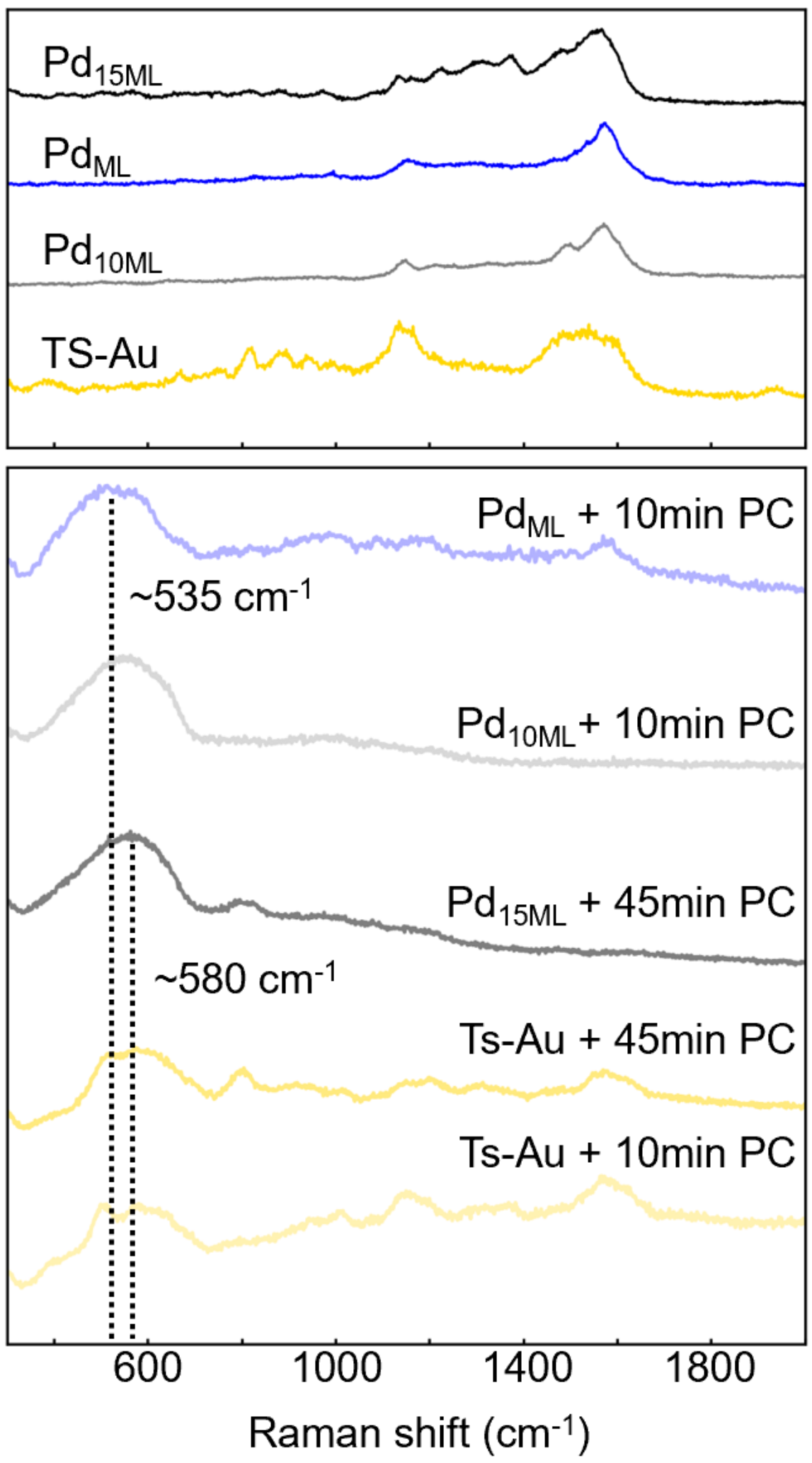

Suppl. Fig. 7| Oxide peak position comparisons. Averaged SERS spectra of Pd films of different thickness, and TS-Au substrates both before (top) and after (bottom) O_2 plasma cleaning. Oxide formed post-cleaning shows distinctly different peak positions near 535 cm^{-1} and 580 cm^{-1}. Multiple peaks around 1500 cm^{-1} are assigned to citrate residues.

Reference

1. J. F. Li, X. D. Tian, S. B. Li, J. R. Anema, Z. L. Yang, Y. Ding, Y. F. Wu, Y. M. Zeng, Q. Z. Chen, B. Ren, Z. L. Wang, Z. Q. Tian, Surface analysis using shell-isolated nanoparticle-enhanced Raman spectroscopy. *Nat. Protoc.* **8**, 52–65 (2013).
2. G. Kang, S. Hu, C. Guo, R. Arul, S. M. Sibug-Torres, J. J. Baumberg, Design rules for catalysis in single-particle plasmonic nanogap reactors with precisely aligned molecular monolayers. *Nat. Commun.* **15**, 9220 (2024).
3. S. Hu, E. S. A. Goerlitzer, Q. Lin, B. De Nijs, V. M. Silkin, J. J. Baumberg, Alchemically-glazed plasmonic nanocavities using atomic layer metals: controllably synergizing catalysis and plasmonics. *Nat. Commun.* **16**, 3370 (2025).
4. Y. Zhang, P. M. Radjenovic, X. Zhou, H. Zhang, J. Yao, J. Li, Plasmonic Core–Shell Nanomaterials and their Applications in Spectroscopies. *Adv. Mater.* **33**, 2005900 (2021).
5. Y. Ding, F. Fan, Z. Tian, Z. L. Wang, Atomic Structure of Au−Pd Bimetallic Alloyed Nanoparticles. *J. Am. Chem. Soc.* **132**, 12480–12486 (2010).
6. L. A. Kibler, M. Kleinert, R. Randler, D. M. Kolb, Initial stages of Pd deposition on Au(hkl) Part I: Pd on Au(111). *Surf. Sci.* **443**, 19–30 (1999).
7. E. A. Weiss, G. K. Kaufman, J. K. Kriebel, Z. Li, R. Schalek, G. M. Whitesides, Si/SiO2-templated formation of ultraflat metal surfaces on glass, polymer, and solder supports: their use as substrates for self-assembled monolayers. *Langmuir ACS J. Surf. Colloids* **23**, 9686–9694 (2007).
8. R. Arul, D.-B. Grys, R. Chikkaraddy, N. S. Mueller, A. Xomalis, E. Miele, T. G. Euser, J. J. Baumberg, Giant mid-IR resonant coupling to molecular vibrations in sub-nm gaps of plasmonic multilayer metafilms. *Light Sci. Appl.* **11**, 281 (2022).
9. D.-B. Grys, M. Niihori, R. Arul, S. M. Sibug-Torres, E. W. Wyatt, B. de Nijs, J. J. Baumberg, Controlling Atomic-Scale Restructuring and Cleaning of Gold Nanogap Multilayers for Surface-Enhanced Raman Scattering Sensing. *ACS Sens.* **8**, 2879–2888 (2023).
10. S. M. Sibug-Torres, D.-B. Grys, G. Kang, M. Niihori, E. Wyatt, N. Spiesshofer, A. Ruane, B. De Nijs, J. J. Baumberg, In situ electrochemical regeneration of nanogap hotspots for continuously reusable ultrathin SERS sensors. *Nat. Commun.* **15**, 2022 (2024).
11. S. Hu, E. Elliott, A. Sánchez-Iglesias, J. Huang, C. Guo, Y. Hou, M. Kamp, E. S. A. Goerlitzer, K. Bedingfield, B. De Nijs, J. Peng, A. Demetriadou, L. M. Liz-Marzán, J. J. Baumberg, Full Control of Plasmonic Nanocavities Using Gold Decahedra-on-Mirror Constructs with Monodisperse Facets. *Adv. Sci.* **10**, 2207178 (2023).
